# Vitexin protects against hypoxic-ischemic injury via inhibiting Ca^2+^/Calmodulin-dependent protein kinase II and apoptosis signaling in the neonatal mouse brain

**DOI:** 10.18632/oncotarget.16065

**Published:** 2017-03-10

**Authors:** Jia-Wei Min, Wei-Lin Kong, Song Han, Nageeb Bsoul, Wan-Hong Liu, Xiao-Hua He, Russell M. Sanchez, Bi-Wen Peng

**Affiliations:** ^1^ Department of Physiology, Hubei Provincial Key Laboratory of Developmentally Originated Disorders, School of Basic Medical Sciences, Wuhan University, Wuhan, Hubei, China; ^2^ Department of Surgery, College of Medicine, Texas A&M Health Science Center, Temple, TX, USA

**Keywords:** c-glycosylated flavonoid, neonatal ischemic, neuroprotection, apoptosis, oxygen-glucose deprivation

## Abstract

Neonatal hypoxic-ischemic is a major cause of death and disability in neonates. In this study, we suggest for the first time that pretreatment with vitexin may suppress a pro-apoptotic signaling pathway in hypoxic-ischemic neuronal injury in neonates by inhibition of the phosphorylation of Ca^2+^/Calmodulin-dependent protein kinase II. Here we found that vitexin pretreatment reduced brain infarct volume in a dose-dependent manner. In addition, vitexin decreased the number of TUNEL-positive cells and brain atrophy. Furthermore, vitexin improved neurobehavioral outcomes. Vitexin also reduced oxygen glucose deprivation-induced neuronal injury and calcium entry. Vitexin pretreatment increased the Bcl-2/Bax protein ratio and decreased phosphorylation of Ca^2+^/Calmodulin-dependent protein kinase II and NF-κB, cleaved caspase-3 protein expression 24 hours after injury. Our data indicate that pretreatment with vitexin protects against neonatal hypoxic-ischemic brain injury and thus has potential as a treatment for hypoxic-ischemic brain injury.

## INTRODUCTION

Neonatal hypoxic-ischemic (HI) is a common cause of death and long-term neurological injuries in newborns [1]. The mechanisms underlying HI brain damage involve excitotoxicity, apoptosis, and inflammation [2]. In this process, due to absence of oxygen reaching the brain, anaerobic glycolysis is rapidly initiated, it will result in an inadequate supply of energy [3]. As result of energy failure, a battery of biochemical events will occur. For example, Glutamate-dependent NMDA receptors will be over-stimulated and intracellular calcium levels will enhanced. At last, overproduction of reactive oxygen species (ROS) and activation of nitric oxide synthase may be leading to excitotoxicity in neuron [4]. It seems that inhibition of Ca^2+^ toxicity may be neuroprotective for HI brain injury.

CaMKII is very important to synaptogenesis and plasticity during brain development [5–7]. When hypoxic-ischemic occur, high level of intracellular Ca^2+^ leads to the binding of Ca^2+^/calmodulin complexes, which will further activate CaMKII [8]. Interestingly, it has been shown that inhibition of CaMKII can improve neural cell survival in neonatal HI [9].

Vitexin (5, 7, 4-trihydroxyflavone-8-glucoside, Vit) is a c-glycosylated flavonoid, which has been found in medicinal and other plants. It has been reported to confirm that Ca^2+^ overload could be inhibited in cardiomyocytes induced by anoxia and reoxygenation with vitexin preconditioning [10]. Vitexin also provides a protection on cardiac hypertrophy through Ca^2+^-mediated calcineurin-NFATc3 and CaMKII signaling pathways [11]. Furthermore, it has been shown that the neuroprotective function of vitexin are closely associated with the inhibition of apoptosis and Ca^2+^ overload in cultured cortical neurons [12]. Previous studies have also found that vitexin reduced hypoxia-ischemia neonatal brain damage in a rat pup model [13] and ischemia/reperfusion injury in adult mice [14]. Therefore, vitexin has the potential to explore the roles of CaMKII *in vitro* and *in vivo*.

In this study, we used primary neuronal cultures and a mouse model of neonatal HI to illuminate that vitexin pretreatment can provide a neuroprotection on neonatal hypoxic-ischemic brain injury. We also demonstrated vitexin can suppress apoptosis in hypoxic-ischemic neuronal injury in neonates by inhibition of CaMKII.

## RESULTS

### Vitexin pretreatment produces a dose-dependent reduction in the infarct volume following hypoxic-ischemic injury

**Figure 1:**
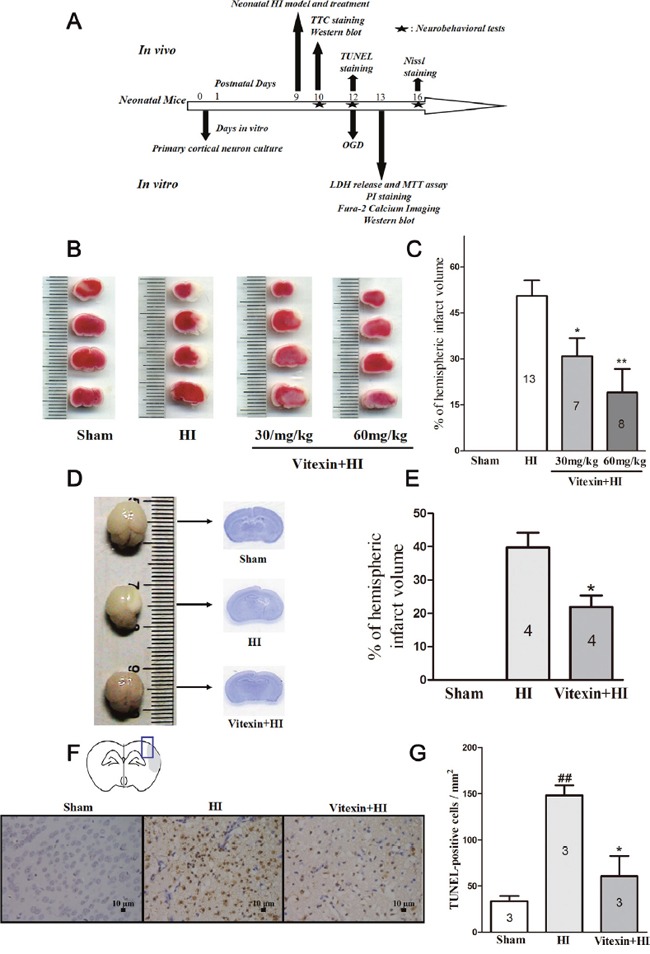
Vitexin (Vit) pretreatment reduces infarct volume in neonatal hypoxic-ischemic (HI) brain injury in a dose-dependent manner and protects against (HI)–induced brain atrophy and neuronal apoptosis **(A)** Diagram of the experimental design. Vitexin was administered intraperitoneally 30 minutes before HI and pre-incubated with cells for 30 minutes prior to oxygen and glucose deprivation (OGD). **(B)** Representative images of 2,3,5-triphenyltetrazolium chloride (TTC)-stained coronal brain slices from sham, HI and treatment groups with different dosages of vitexin are shown. **(C)** Quantitative analysis of infarct volume revealed that vitexin treatment produced a dose-dependent reduction in infarct volume (HI, 50.57 ± 5.05%, n =13; Vitexin (30mg/kg) +HI, 30.86 ± 5.92%, n =7; Vitexin (60mg/kg) +HI, 19.01 ± 7.72%, n =8). **(D)** Representative images of whole brains and Nissl-stained brain slices. **(E)** Quantitative analysis of infarct volume revealed a reduction of infarct volume in the vitexin (60 mg/kg)-treated group (HI, 39.75 ± 4.46%, n =4; Vitexin + HI, 21.92 ± 3.42% n =4). **(F)** Images showed representative TUNEL-positive cells. **(G)** Quantitative analysis of TUNEL-positive cells of the different groups. The TUNEL-positive cells were counted per ×40 field in the penumbra area. Vitexin pretreatment reduced the number of TUNEL-positive cells (sham, 33.67 ± 5.55 cells, n=3; HI, 148.10 ± 11.17 cells, n=3; Vitexin + HI, 60.65 ± 21.73 cells, n=3). Data represent the means ± SEM of three independent experiments. The significance of difference between means was analysed by the ANOVA and Tukey's post hoc test (for multiple comparisons) and by the Student's t-test (for single comparisons) (* *P*<0.05 versus HI; **P<0.01 versus HI; ## *P*<0.01 versus sham).

Quantitative assessment of TTC-stained sections indicated that vitexin pretreatment (30 and 60 mg/kg) significantly attenuated the infarct volume to 30.86 ± 5.92% (*P*<0.05) and 19.01 ± 7.72% (*P*<0.01), respectively, compared to that of the HI group, which was 50.57 ± 5.05%. Vitexin pretreatment reduced infarct volume in a dose-dependent manner (Figure 1B and 1C). For the remaining experiments, we used 60 mg/kg dose to elucidate the molecular mechanisms.

### Vitexin inhibits HI-induced brain atrophy and neuronal apoptosis

Vitexin (60 mg/kg) also showed less brain damage compared with the HI group (which had greater brain tissue loss and atrophy) (Figure 1D and 1E). Moreover, reducing apoptosis is also good for neonatal stroke. Therefore, to determine whether vitexin pretreatment reduced apoptosis, we performed a TUNEL assay 3 days post- HI insult. The results showed that the number of TUNEL-positive cells had been notably decreased with vitexin pretreatment (60.65 ± 21.73 cells; *P*<0.05) in the peri-infarct region of the compared with the HI group (148.10 ± 11.17 cells versus 33.67 ± 5.55 cells in the sham group; *P*<0.01) (Figure 1F and 1G). This result indicated that vitexin pretreatment inhibited HI-induced apoptosis.

### Vitexin improves neurological outcomes after HI

**Figure 2:**
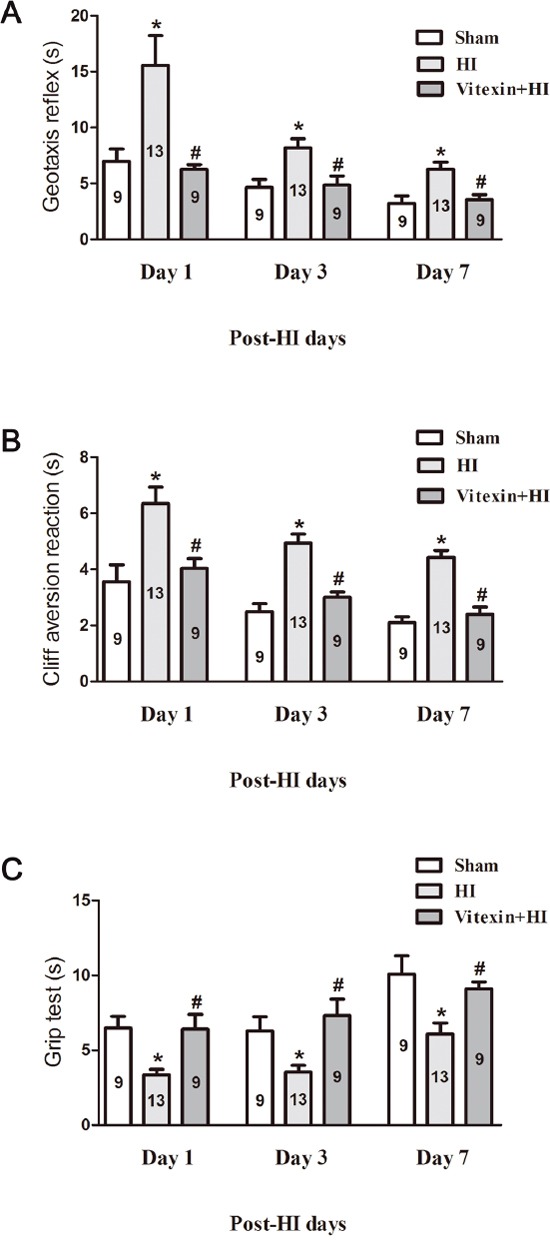
Vitexin pretreatment improves neurobehavioral performance after cerebral HI Neurobehavioral evaluation was performed as described in the Materials and Methods. **(A)** Geotaxis reflex (sham, 1 day: 6.99 ± 1.08 s; 3 days: 4.64 ± 0.72 s; 7 days: 3.22 ± 0.67 s, n=9; HI, 1 day: 15.55 ± 2.67 s; 3 days: 8.19 ± 0.81 s; 7 days: 6.27 ± 0.63 s, n=13; Vitexin+HI, 1 day: 6.26 ± 0.42 s; 3 days: 4.84 ± 0.83 s; 7 days: 3.55 ± 0.44 s, n=9). **(B)** Cliff aversion reaction (sham, 1 day: 3.56 ± 0.60 s; 3 days: 2.49 ± 0.29 s; 7 days: 2.10 ± 0.21 s, n=9; HI, 1 day: 6.35 ± 0.58 s; 3 days: 4.93 ± 0.33 s; 7 days: 4.43 ± 0.25 s, n=13; Vitexin+HI, 1 day: 4.04 ± 0.35 s; 3 days: 3.01 ± 0.18 s; 7 days: 2.39 ± 0.26 s, n=9), and **(C)** Grip test (sham,1 day: 6.48 ± 0.80 s; 3 days: 6.30 ± 0.94 s; 7 days: 10.09 ± 1.22 s, n=9; HI, 1 day: 3.35 ± 0.37 s; 3 days: 3.55 ± 0.45 s; 7 days: 6.09 ± 0.74 s, n=13; Vitexin+HI, 1 day: 6.40 ± 0.99 s; 3 days: 7.32 ± 1.09 s; 7 days: 9.10 ± 0.47 s, n=9) were measured 1 day, 3 days and 7 days after HI in the different groups. Data represent the means ± SEM of three independent experiments. The significance of difference between means was analysed by the ANOVA and Tukey's post hoc test (for multiple comparisons) (* *P*<0.05 versus sham; # *P*<0.05 versus HI).

We next determined whether vitexin improved neurological performance by assessing the animals' performance on a series of tests, including a geotaxis reflex test, a cliff avoidance reaction test, and a grip test. Tests were performed in the sham, HI and vitexin-treated HI groups at 1, 3, and 7 days after HI. In the geotaxis reflex test, mice in the sham group (1 day: 6.99 ± 1.08 s; 3 days: 4.64 ± 0.72 s; 7 days: 3.22 ± 0.67 s) exhibited a significantly shorter latency to complete the reflex than those in the HI group (1 day: 15.55 ± 2.67 s; 3 days: 8.19 ± 0.81 s; 7 days: 6.27 ± 0.63 s). Mouse pups in the HI group exhibited a significant longer latency at 1 day, 3 days and 7 days after the insult compared to those in the vitexin-treated group (1 day: 6.26 ± 0.42 s; 3 days: 4.84 ± 0.83 s; 7 days: 3.55 ± 0.44 s) (Figure 2A, *P*<0.05). In the cliff avoidance reaction test, the mice in the sham group (1 day: 3.56 ± 0.60 s; 3 days: 2.49 ± 0.29 s; 7 days: 2.10 ± 0.21 s) required significantly less time to respond to the cliff than the mice in the HI group (1 day: 6.35 ± 0.58 s; 3 days: 4.93 ± 0.33 s; 7 days: 4.43 ± 0.25 s.). The mice treated with vitexin (1 day: 4.04 ± 0.35 s; 3 days: 3.01 ± 0.18 s; 7 days: 2.39 ± 0.26 s) showed a significant reduction in latency at 1 day, 3 days and 7 days compared with the HI group (Figure 2B, *P*<0.05). In the grip test, the mice in the HI group reduced grip ability (1 day: 3.35 ± 0.37 s; 3 days: 3.55 ± 0.45 s; 7 days: 6.09 ± 0.74 s) compared with the sham group (1 day: 6.48 ± 0.80 s; 3 days: 6.30 ± 0.94 s; 7 days: 10.09 ± 1.22 s). Vitexin pretreatment improved the grip ability of the mice (1 day: 6.40 ± 0.99 s; 3 days: 7.32 ± 1.09 s; 7 days: 9.10 ± 0.47 s) compared with the HI group (Figure 2C, *P*<0.05). Therefore, these data indicated that pretreatment with vitexin restored behavioral outcomes after HI challenge.

### Neuroprotective effects of vitexin pretreatment against OGD-induced neuronal injury

**Figure 3:**
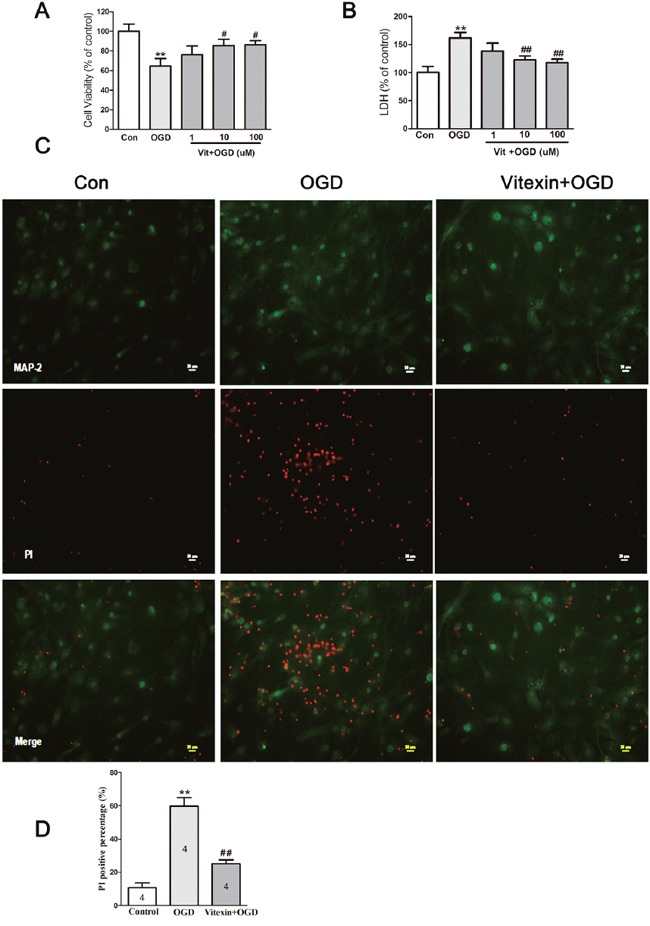
Vitexin pretreatment promotes neuronal survival under OGD **(A)** Cells that were pretreated with vitexin exhibited a dose-dependent reduction in OGD-induced neuronal injury, as demonstrated by increased 3-(4,5-dimethylthiazol-2-yl)-2,5-diphenyltetrazolium (MTT) levels in cell lysates at 24 hours of reoxygenation (control, 100 ± 7.47%, n=10; OGD, 64.65 ±7.67%, n=10; Vit (1μmol/L) + OGD, 76.34 ± 8.69%, n=10; Vit (10μmol/L) + OGD, 85.47 ± 6.52%, n=10; Vit (100μmol/L) + OGD, 86.33 ± 4.15% n=10). **(B)** Vitexin pretreatment also reduced lactate dehydrogenase (LDH) levels in the culture media (control, 100 ± 11.03%, n=10; OGD, 161.8±9.59%, n=10; Vit (1μmol/L) + OGD, 138.2 ± 14.19%, n=10; Vit (10μmol/L) + OGD, 123.1 ± 6.47%, n=10; Vit (100μmol/L) + OGD, 117.4 ± 6.47%, n=10). **(C)** Representative images of propidium iodide (PI) staining. Scale bar represents 20 μm. **(D)** Quantitative analysis of PI-positive cells in the different groups. The PI-positive cells were counted per ×20 field. Pretreatment with vitexin (10 μmol/L) reduced the percentage of PI-positive cells (control, 10.75 ± 2.81%, n=4; OGD, 59.75 ± 5.23%, n=4; Vit (10μmol/L) + OGD, 25.00 ±2.48%, n=4). Data represent the means ± SEM of three independent experiments. The significance of difference between means was analysed by the ANOVA and Tukey's post hoc test (for multiple comparisons) (** *P*<0.01 versus control; # *P*<0.05, ## *P*<0.01 versus OGD).

In the primary neurons under the OGD model [31], MTT levels decreased (Figure 3A) and LDH release increased at 24 hours of reoxygenation (Figure 3B) compared to control levels. Vitexin pre-incubation for 30 minutes significantly improved OGD-treated cell survival and reduced LDH release in a concentration-dependent manner. Compared with the cell viability in the OGD group (64.65 ±7.67%), cell viability significantly increased to 76.34 ± 8.69%, 85.47 ± 6.52%, and 86.33 ± 4.15% for 1, 10, and 100 μmol/L of vitexin, respectively (** *P*< 0.01 vs control, # *P*< 0.05 vs OGD). LDH release was also markedly decreased by vitexin (1, 10, and 100 μmol/L) compared to that in the OGD group (** *P*<0.01 vs control, ## *P*<0.01 vs OGD). Considering that the greatest neuroprotective effects of vitexin were observed at 10 μmol/L, this concentration would be better used in the following vitro studies. Vitexin pretreatment protected against OGD-induced neuronal injury were further confirmed by PI staining. As shown in Figure 3C and 3D, the percentage of PI-positive cells was 10.75 ± 2.81% in the no OGD-treated group, and this increased significantly to 59.75 ± 5.23% in the OGD group (** *P*<0.01) but was significantly decreased in the vitexin pretreatment group (25.00 ±2.48%; 10 μmol/L; ## *P*<0.01). These data indicated that vitexin may be neuroprotective to OGD-treated cells.

### Vitexin reduces the intracellular Ca^2+^ concentration ([Ca^2+^]_i_) in OGD-treated cells and inhibits the apoptosis proteins expression via the CaMKII/NF-κB pathway

**Figure 4:**
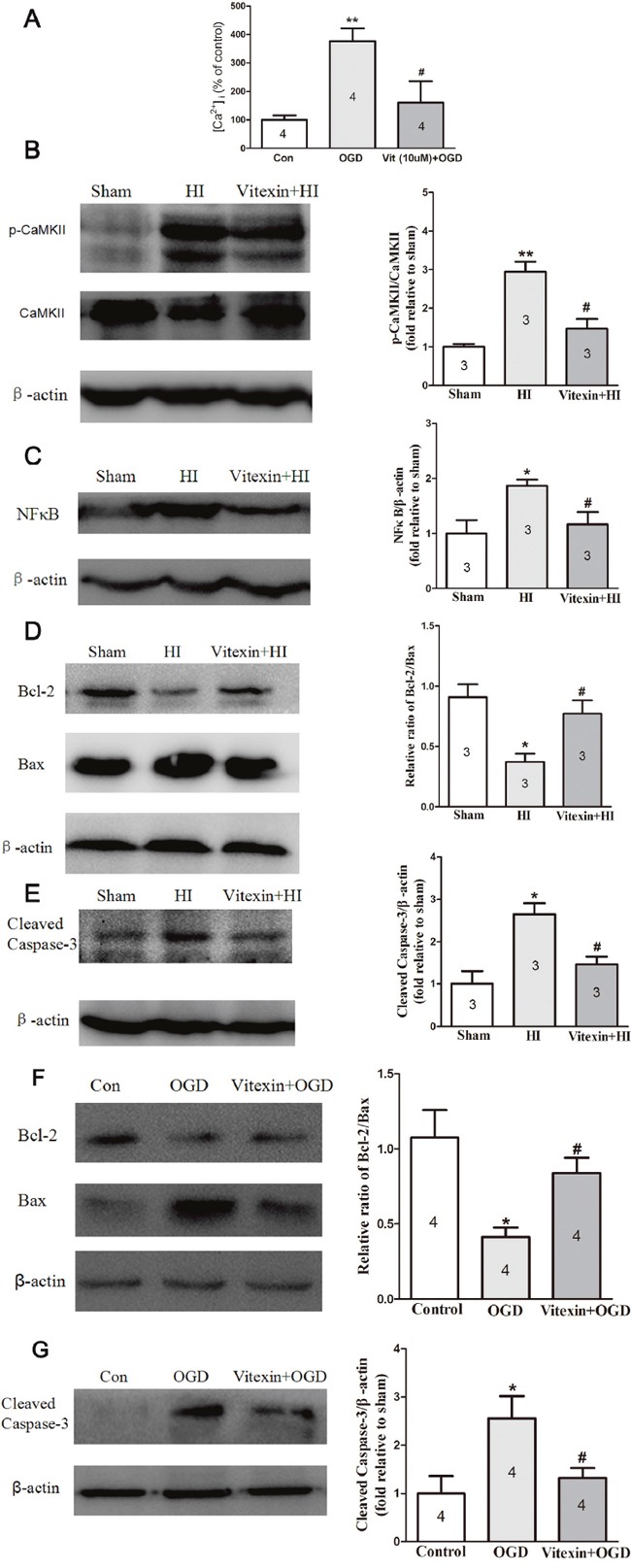
Vitexin pretreatment reduces ([Ca2+]i) in cells after OGD and inhibits CaMKII, NF-κB and apoptosis-related proteins expression *in vivo* and *in vitro* post-HI insult **(A)** Vitexin decreased the effects of OGD in primary cortical neurons, resulting in an increase in [Ca^2+^]_i_ (control, 100 ± 16.12%, n=4; OGD, 376.8 ± 44.27%, n=4; Vit (10μmol/L) + OGD, 160.4 ± 5.32%, n=4). **(B)** Representative Western blots showing the effects of vitexin on CaMKII expression and the phosphorylation status of CaMKII 1 day after HI. Vitexin (60 mg/kg) pretreatment inhibited HI-induced CaMKII phosphorylation (sham, 1.00 ± 0.07, n=3; HI, 2.94 ± 0.26, n=3; Vitexin+HI, 1.47 ± 0.25, n=3). **(C)** Representative Western blots showing the effects of vitexin on nuclear NF-κB expression 24 hours after HI. Vitexin pretreatment decreased NF-κB expression compared to that in the HI group (sham, 1.00 ± 0.24, n=3;. HI, 1.87± 0.11, n=3; Vitexin+HI, 1.17± 0.22, n=3). **(D)** Representative Western blot showing that vitexin pretreatment increased the ratio of Bcl-2/Bax protein (sham, 0.91 ± 0.11, n=3; HI, 0.37 ± 0.07, n=3; Vitexin+HI, 0.77 ± 0.11, n=3). **(E)** Decreased cleaved caspase-3 protein expression 1 day after HI (sham, 1.00 ± 0.30, n=3; HI, 2.65 ± 0.26, n=3; Vitexin+HI, 1.45 ± 0.19, n=3). **(F)** TheBcl-2/Bax ratio in the OGD group was lower than that in the control group and was significantly increased in the vitexin (10 μmol/L) pretreatment group (control, 1.08 ± 0.18, n=3; HI, 0.41 ± 0.06, n=3; Vitexin+HI, 0.84± 0.10, n=3). **(G)** Vitexin pretreatment also decreased the expression of cleaved caspase-3 (control, 1.00 ± 0.36, n=3; HI, 2.57 ± 0.46, n=3; Vitexin+HI, 1.32± 0.20, n=3). Data represent the means ± SEM of three independent experiments. The significance of difference between means was analysed by the ANOVA and Tukey's post hoc test (for multiple comparisons) (* *P*<0.05, ** *P*<0.01 versus sham; # *P*<0.05 versus HI).

The neuronal death induced by OGD is mainly related to calcium overload [15]. So, calcium signals in cultured cortical neurons have been measured by using the fluorescent Ca^2+^ indicator Fura-2-AM. As shown in Figure 4A, OGD-treated cortical neurons resulted in an increase in [Ca^2+^]_i_ (** *P*<0.01). However, vitexin reduced this increase (# *P*<0.05).

Neural Ca^2+^ binds to calmodulin, forming a Ca^2+^/ calmodulin complex, which can activate CaMKII through its autophosphorylation. Furthermore, inhibition of CaMKII may provide the neuroprotection [16, 17]. We then compared the expression of p-CaMKII/CaMKII in brain tissue ipsilateral to the occlusion that was collected from each group. As shown in Figure 4B, p-CaMKII/CaMKII expression was significantly increased 24 hours after HI (2.94 ± 0.26-fold of sham; *P*<0.01). Vitexin significantly reduced the p-CaMKII/CaMKII expression (1.47 ± 0.25-fold of sham; *P*<0.05). The inhibition of NF-κB has been shown to be protective in the developing brain after neonatal HI injury [18]. Therefore, we examined the level of NF-κB in brain tissue ipsilateral to the occlusion in the different groups. We found that NF-κB protein expression was increased in the HI group (1.87± 0.11-fold of sham; *P*<0.05) and the increase was attenuated by pretreatment with vitexin (1.17± 0.22-fold of sham; *P*<0.05) (Figure 4C). The TUNEL staining data indicated that vitexin may attenuate apoptosis in neonatal HI brain injury. Because the Bcl-2/Bax ratio and caspase-3 are primary indicators of apoptosis, we further determined the levels of these proteins in brain tissue ipsilateral to the occlusion and compared the levels between the three different groups. The ratio of Bcl-2/Bax protein was significantly decreased in the HI group (0.37 ± 0.07; *P*< 0.05) compared with the sham group (0.91 ± 0.11), while the ratio of Bcl-2/Bax protein was almost equivalent to the sham group level in the vitexin-treated group (0.77 ± 0.11; *P*< 0.05) (Figure 4D). Similarly, the expression of cleaved caspase-3 protein significantly increased post-HI (2.65 ± 0.26-fold of sham; *P*<0.05). Vitexin pretreatment significantly reduced the cleaved caspase-3 protein level after HI (1.45 ± 0.19-fold of sham; *P*<0.05) (Figure 4E). Next, the effects of vitexin on the Bcl-2/Bax ratio and cleaved caspase-3 expression were measured in the OGD-treated cortical neuron. As shown in Figure 4F and 4G, cortical neurons exposed to OGD had a lower Bcl-2/Bax ratio and higher cleaved caspase-3 expression, while the Bcl-2/Bax ratio was increased and cleaved caspase-3 expression was decreased with the vitexin (10 μmol/L) treatment (*, #, *P*<0.05). All of the data demonstrated that anti-apoptotic effects contribute to the neuroprotection of vitexin via the CaMKII/NF-κB pathway.

## DISCUSSION

Perinatal hypoxic-ischemic encephalopathy is a primary cause of morbidity and mortality in newborns. In this study, we suggest for the first time that pretreatment with vitexin may inhibit neuronal apoptosis in neonatal hypoxic-ischemic injury by suppression of CaMKII. First, TTC staining data showed that vitexin pretreatment attenuated brain damage 24 hours after HI. Whole-brain imaging and Nissl staining also confirmed its neuroprotective effect. Furthermore, vitexin significantly decreased the amount of TUNEL-positive neuronal cells 3 days post-HI. Neonatal hypoxic-ischemic brain injury leads to functional recovery loss [19]. Therefore, to evaluate whether vitexin alters animal functional recovery, we assessed the neurobehavioral test in each group. Vitexin improved functional recovery in these tests. So, our data indicated that vitexin ameliorated brain injury and improved functional recovery after HI. Next, we also found that vitexin pretreatment improved the OGD-treated cells survival. Our findings indicated that vitexin may be neuroprotective both *in vitro* and *in vivo* after HI insult.

Anoxic or ischemic neuronal death always causes Ca^2+^ toxicity [20–22]. It initiates a battery of cytoplasmic and nuclear events, containing apoptotic pathway [23]. Ischemic-hypoxic can elevate calcium level through a number of voltage-gated and ligand-gated calcium channels [24]. Yang et al found vitexin could reduce the calcium influx in cultured cortical neurons via inhibiting the activities of NR2B-containing NMDA receptors [12]. Similarly, we also found that vitexin reduced the intracellular Ca^2+^ concentration ([Ca^2+^]_i_) in OGD-treated cells. However, since vitexin has lots of cellular targets that contribute to its neuroprotective effects, further studies need to be done to identify which calcium channel that is sensitive to vitexin.

CaMKII activation will occur when intracellular Ca^2+^ is increased, leading to the binding of Ca^2+^/calmodulin complexes [8]. Previous studies have shown that increases in CaMKII activity resulted in cell death in several different animal models. Such as: Gao et al demonstrated that CaMKII contributes to neural death through the phosphorylation of acid-sensing ion channels (ASICs) in ischemic stroke rat [25]. Vitexin provided its antihypertrophic effect via interruption of the Ca^2+^–CaMKII pathways [11]. Similarly, in our present study, vitexin inhibited the high levels of phospho-CaMKII, indicating that de-phosphorylated CaMKII may contribute to the neuroprotective effects of vitexin. CaMKII has been implicated in the activation of NF-κB, NF-κB can activate iNOS expression and then accumulate huge lots of NO [26]. Our results found the p65 subunit of NF-κB can be activated and transferred to nuclei in cortical tissue after HI and this effect could be attenuated by vitexin pretreatment. An increase in oxidants may lead to activate p38 and JNK [27]. JNK mediates related to the pro-apoptotic proteins Bax and Bak [28]. Western blots revealed that the ratio of Bcl-2/Bax protein was higher in vitexin-treated group. Vitexin also upregulated Bcl-2/Bax and Caspase-3 expression in OGD-treated cells, which has been identified as a key mediator of apoptosis in ischemic stroke pups [29]. The data showed that vitexin precondition markedly inhibited cleaved caspase-3 expression. Similarly, vitexin also decreased cleaved caspase-3 expression in OGD-treated cells. It indicated that inhibiting activation of cellular apoptotic pathways may contribute to the neuroprotective effect of vitexin against HI in neonatal mice.

In conclusion, vitexin pretreatment protected the brain, improved functional recovery, promoted survival and inhibited apoptosis signaling pathways in hypoxic-ischemic brain damage. We also found that the protective role and potential therapeutic value of vitexin for treating neonatal HI brain injury was associated with inhibition of CaMKII activity and anti-apoptotic mechanisms. But, it is noted that vitexin has a plenty variety of cellular targets that contribute to its neuroprotective effects, identifying the specific target of vitexin should be addressed in the future.

## MATERIALS AND METHODS

### Materials

Vitexin (Tauto Biotechnology Co., Ltd, Shanghai, China), dimethyl sulfoxide (DMSO), and poly-D-lysine were purchased from Sigma-Aldrich, USA. Neurobasal™ medium, B27 supplement, fetal bovine serum (FBS) and other cell culture materials were purchased from Gibco Life Technologies Corporation USA. All other reagents used were purchased from Sigma-Aldrich, USA unless stated otherwise.

### Animal experiments

C57BL/6 mice were obtained from the Wuhan University Center for Animal Experiment/ABSL-3 Laboratory. The protocol was approved by the Committee on the Ethics of Animal Experiments of Wuhan University (China) (Permit Number: SCXK 2008-0004). Mouse HI was carried out according to a method described previously [13, 30]. Briefly, postnatal day (P9) C57BL/6 mice of both sexes were anesthetized by inhalation of diethyl ether. The right common carotid artery (CCA) was exposed and ligated. The mice were then returned to their dam and allowed to recover for 1.5 hours. They were then placed in an airtight, transparent chamber partially submerged in a 37°C water bath to maintain a constant thermal environment and subjected to 10% O_2_ in N_2_ for 40 minutes. Thereafter, animals were returned to their mothers. Sham group animals underwent anesthesia and neck incision only.

### Drug administration

The experimental design is illustrated in Figure 1A. Ninety-five mouse pups were randomly assigned to one of the following groups: sham group, HI group or vitexin+HI (Vit, 30 and 60 mg/kg) group. Vitexin was dissolved in normal saline. An equivalent normal saline and vitexin solution were administered intraperitoneally 30 minutes before the induction of HI. The same amount of normal saline was used in the sham group.

### Primary cortical neuron culture

Primary cortical neuronal cultures were prepared from the cerebral cortices of ten neonatal C57BL/6 mice (postnatal of 0–1) as described previously [31]. Briefly, primary cortical neurons were grown in poly-D-lysine-coated 96- 24- or 6-well plates, which consisted of Neurobasal™ medium, 2% B27 supplement, 2-mmol/L L-glutamine, and 1% penicillin–streptomycin. At 3 days *in vitro* (DIV), one-third of the media was replaced with fresh medium (without L-glutamine) containing cytosine arabinofuranoside (AraC, 5 μmol/L) to kill the growth of non-neuronal cells. OGD experiments were conducted at 12 DIV, when cultures consisted mainly of neurons (>95% MAP-2 immunoreactive cells) (MAP-2; Abcam, Cambridge, MA USA).

### Oxygen-glucose deprivation

To induce oxygen-glucose-deprived conditions, cultured cortical neurons at 12 DIV were exposed to OGD as described previously [31]. Briefly, cultured cortical neurons were incubated with vitexin in glucose-free and oxygen-deprived DMEM for 30 minutes, followed by incubation in an anaerobic chamber flushed with 5% CO_2_ and 95% N_2_ (v/v) at 37°C for 2 hours. At the end of this period, the cells were removed from the hypoxic chamber to a regular incubator (5% CO_2_ and 21% O_2_) and cultures were replaced with Neurobasal™ media for 24 hours recovery. The control cells were changed to DMEM with glucose and remained in a regular incubator (5% CO_2_ and 21% O_2_) for the same duration.

### Infarct volume evaluation and general histology

There were thirty-four postnatal day (P9) C57BL/6 mice for TTC staining and twelve neonatal mice for Nissl staining. TTC (2,3,5-triphenyltetrazolium chloride monohydrate) staining was used to measure infarct volume as previously described [13]. Briefly, at 24 hours post-HI, animals were perfused transcardially with PBS under deep anesthesia. The brains were removed and sectioned into 2 mm slices, then immersed into 2% TTC solution at 37 °C for 10 minutes, followed by 4% paraformaldehyde. The infarct volume was traced and analyzed by Image J software (NIH).

Whole brains were dissected 7 days after HI and fixed in 4% paraformaldehyde overnight. Brain tissues also were sectioned coronally for Nissl staining. Briefly, brain slices of 20 μm were stained with 1% cresyl violet for 2 minutes and rinsed quickly in distilled water. Representative images were captured using a camera (Olympus, Japan) in the same field [32].

### TUNEL stain

Nine postnatal day (P9) C57BL/6 mice were used in TUNEL staining. Terminal transferase-mediated dUTP nick-end labeling (TUNEL) staining was performed according to the manufacturer's instructions (Roche).

### Cell counting

For each animal, three coronal section (10 sections apart) was used for TUNEL staining. Six visual fields of the peri-infarct region in cerebral cortex were photographed in each section. The total number of TUNEL-positive cells in each field was counted by the Imaging-Pro-Plus at higher magnification (×40). The data were represented as the total number of cells per mm^2^ [13].

### Neurobehavioral tests

All neurobehavioral evaluations were performed in a blind manner. 1, 3 and 7 days after HI, thirty-one mouse pups were randomly assigned to one of the following groups: sham group, HI group or vitexin+HI group. Animals were evaluated with 3 neurobehavioral tests. These tests evaluated: 1) the geotaxis reflex [33] to evaluate vestibular and/or proprioceptive functions. Animals were placed head down in the middle of an inclined 30 cm board (angle of 30°). The latency to make a 180° turn was recorded up to a maximum time of 60 seconds and each mouse was tested 3 times. 2) The cliff avoidance reaction [34] to check for the presence of maladaptive impulsive behavior. The apparatus consisted of a wood board (50 × 25 × 2.5 cm, L × W × H), with one end protruding 1.5 cm over the edge of a desk. Animals were gently placed on the protruding end of the board with their head down and forepaws off the board, and the latency to place both their forepaws back on the board was measured up to a maximum time of 60 s and each mouse was tested 3 times and 3) grip ability [35] to assess grip force and fatigability. Animals were suspended by both forepaws on a metallic rope (diameter, 1.5 mm) stretched horizontally 50 cm over a cotton pad. Time before falling was recorded (maximum: 60 seconds) and each mouse was tested 3 times.

### Assessment of cell viability and cytotoxicity

Cell viability was monitored using the 3-(4,5-dimethylthiazol-2-yl)-2,5-diphenyltetrazolium (MTT) colorimetric assay. MTT (20 μL) was added to the cell culture medium, and the cells were incubated at 37°C for 4 hours. Then, after the medium was discarded, 200 μL DMSO was added, and absorbance at 570 nm was recorded. Cell cytotoxicity was assessed by measuring the amount of lactate dehydrogenase (LDH) released into the culture medium using a cytotoxicity detection kit (Promega G1780) following the manufacturer's procedure. Briefly, 50 μL of culture medium was mixed with 50 μL substrate and incubated in a dark place at 37°C for 30 minutes. Then, 50 μL stop solution was added, and the intensity was measured at 490 nm [36].

### PI staining

OGD-induced cell injury was determined by propidium iodide (PI) staining images [32]. Briefly, cells were stained with PI for 30 minutes and then fixed with 4% paraformaldehyde in PBS for 30 minutes at RT. After the cells were washed with PBS 3 times, they were incubated overnight with an anti-MAP-2 antibody (1:1000) and subsequently incubated with a fluorescein isothiocyanate (FITC)-conjugated anti-rabbit secondary antibody (1:100) for 2 hours at room temperature. Images were taken using a microscope. For each group, four wells were used for PI staining. Six visual fields of the stained cells were photographed in each well. The total number of stained cells was counted using Imaging-Pro-Plus at higher magnification (×20). The percentage of PI-positive cells (%) = (PI positive cell number/total cell number)*100%.

### Measurements of intracellular Ca^2+^ concentration ([Ca^2+^]_i_)

The [Ca^2+^]_i_ in OGD-treated primary cortical neurons were determined using the fluorescent Ca^2+^ indicator Fura-2-AM [37]. Briefly, primary cortical neurons were incubated with 2μM fura-2 AM after 24 hours OGD for 45 minutes at 37 °C on the shaker. Neurons were exposed to 1 mM MnCl_2_ in Ca^2+^-free HEPES–MEM and were excited every 10 seconds at 340 and 380 nm, and the emission fluorescence at 505 nm was recorded. The Ca^2+^-insensitive fluorescence was subtracted from each wavelength before calculations. The MnCl_2_-corrected 340/380 emission ratios were converted to concentration using the Grynkiewicz [38] equation as follows: [Ca^2+^]_i_=*K*d×[(R-R_min_)/(R_max_-R)]×β, where *K*d was 224 nmol/L and R(F340/F380) was the fluorescence intensity. R_max_ was determined by adding Triton X-100 (final concentration of 0.1%), R_min_ was determined by adding EGTA (final concentration of 5 mmol/L), and β is the ratio of the 380 nm fluorescence intensity of R_min_ to that of R_max_ [39].

### Western blots

Proteins extracted from brain tissues (three pups for each group) collected 24 hours after HI and primary cortical neuron cultures 24 hours after OGD were used for Western blot analysis. The Western blotting experiments were carried out as described previously [26]. Briefly, the animals were euthanised with a single intraperitoneal injection of 2ml of sodium pentobarbitone (200mg/ml). The brain samples were collected. Proteins from the ipsilateral hemisphere and primary cortical neurons were extracted by homogenizing in RIPA buffer (Santa Cruz Biotechnology, Santa Cruz, CA), and further centrifuged at 12,000 g at 4°C for 15 minutes. The supernatant was used as whole cell protein extract and the protein concentration was determined by using a detergent compatible assay (Bio-Rad, Dc protein assay). Equal amounts of protein were loaded on an SDS-PAGE gel. After being electrophoresed and transferred to a polyvinylidene fluoride membrane, the membrane was blocked by immersion for 3 hours in Tris-buffered saline (TBS) containing 5% dry milk and incubated with the primary antibody overnight at 4 °C.

The primary antibodies used were mouse anticapsaicin receptor antibodies (1:500; Millipore Corporation, USA), rabbit polyclonal anti-cleaved caspase-3 (1:500), Bcl-2 (1:1000), Bax (1:500), anti-CaMKII (1:200), anti-phospho-CaMKII (Thr286) (1:200; Cell Signaling Technology, Danvers, Mass), anti-NF-κB p65 (1:100; Santa Cruz, CA, USA) and mouse monoclonal anti-β-actin (Tianjin Sungene Biotech Co. Ltd, Tianjin, China, KM9001, 1:2000). After incubation, the membranes were washed at least three times with TBST (TBS containing 0.2% Tween-20) and were then incubated again for 1.5 hours with the goat anti-mouse IgG for TRPV1 and β-actin (diluted 1: 20,000), goat anti-rabbit IgG for cleaved caspase-3, Bcl-2, Bax, CaMKII, phospho-CaMKII and NF-κB p65 (diluted 1: 20,000) at room temperature. The membranes were washed again with TBST three times. Finally, the reaction was developed using a chemiluminescent reagent (ECL; Ecl Advantage Inc., Menlo Park, California, USA) and exposed to Hyper film (GE Healthcare Life Sciences, Pittsburgh, Pennsylvania, USA). The data were analyzed by the software Quantity one 4.6.1 (Bio-Rad).

### Statistical analysis

All experiments were performed blindly and were repeated at least three times. Statistical analysis was conducted using the PRISM 5.0 (GraphPad Software, La Jolla, CA, USA). Data were expressed as the mean ± SEM. For statistical analysis, non-parametric tests were used. Student's t-test was used for comparison between two groups, and one-way ANOVA followed by a Tukey's post hoc test for comparison between more than two groups. *P*<0.05 was considered to be statistically significant.

## Abbreviations

HI: hypoxic-ischemic
LDH: lactate dehydrogenase
MAP2: microtubule-associated protein 2
OGD: oxygen–glucose deprivation
TUNEL: terminal dexynucleotidyl transferase-mediated deoxyuridine triphosphate nick end labeling
CaMKII: calcium/calmodulin dependent protein kinase II
p-CaMKII: phospho-calcium/calmodulin dependent protein kinase II
NF-κb: nuclear factor-Kappa b
Vit: vitexin
DMSO: dimethyl sulfoxide
FBS: fetal bovine serum
CCA: common carotid artery
DIV: days *in vitro*
PI: propidium iodide
FITC: fluorescein isothiocyanate
TTC: 2,3,5-triphenyltetrazolium chloride monohydrate
